# Bibliographical

**Published:** 1867-04

**Authors:** 


### BIBLIOGRAPHICAL.

---

*The Functions and Disorders of the Reproductive Organs in Children, Youth, Adult Age, and Advanced Life; considered in their Physiological, social, and moral relations.* By William Acton, M. R. C. S., &c., &c.

The above work, from the publishing house of Lindsay & Blakiston, Philadelphia, is, we think, a most excellent production. It comprises subjects of the highest importance to the practicing physician, the study of which many have almost entirely neglected. Grave functional disorders of the reproductive organs, producing the most distressing mental and general nervous disturbance, are of such frequent occurrence, and so often lightly regarded, or entirely overlooked by the practitioner, that the thorough study of the subjects contained in the volume before us, is decidedly important to the successful treatment of many forms of mysterious nervous derangement. Patients themselves require to be carefully instructed in many of the principles laid down in the book. Vicious habits, in the voluntary departure from the proper use and management of the organs, often result in the most depraved and wretched condition to which mankind is subject.

The work is a handsome volume, of convenient size, and will be a useful addition to the library of all physicians. Practical works, such as this, which meet the demands of the busy practitioner, are of much more importance to him than large volumes of fine-spun theories on subjects not of immediate and constant interest.

---

We have just received from the author, a pamphlet of 130 pages, on Spurious Vaccination. By Joseph Jones, M. D., Professor of Physiology and Pathology in the Medical Department of the University of Nasville.

The work is made up of Researches upon the "abnormal phenomena accompanying and following vaccination in the Confederate Army."

We have merely glanced at its contents, and cannot, therefore, speak of it only as the researches of a laborious investigator, on a very interesting and important subject.

We expect useful information in its perusal, and shall avail ourselves of it soon.

---

*Injuries of the Spine, with an Analysis of nearly four hundred cases.* By John Ashhurst, jr., A. M., M. D., Fellow of the College of Physicians of Philadelphia; Member of the Academy of Natural Sciences; Member of the Pathological Society of Philadelphia, etc., etc. Philadelphia: J. B. Lippincott & Co. London: Trubuer & Co. 1867.

This beautiful little monograph of 127 pages, embraces all that is known of Injuries of the Spine. There is a large amount of valuable information in a small compass. The Appendix of cases in tabular form, with analysis, embraces 394 cases. Every Physician should have this little volume.

